# Cholecystectomies in the shadow of COVID-19 pandemic: a retrospective analysis of 1075 patients – shift in patient behavior, hospital logistics, and perspectives for the future

**DOI:** 10.1186/s12893-025-03445-z

**Published:** 2025-12-23

**Authors:** Ahmed Abdelsamad, Eric Zahedani, Illya Slobodkin, Ilgar Aghalarov, Tim Fahlbusch, Waldemar Uhl, Mohammed Khaled Mohammed, Andrea Tannapfel, Torsten Herzog

**Affiliations:** 1 Department of Surgery II, Witten/Herdecke University, 58455 Witten, Germany; 2 Department of Surgery, Knappschaft Klinikum Vest, Recklinghausen, 45657 Germany; 3 Department of Surgery, St. Josef Hospital, Ruhr University Bochum, Bochum, Germany; 4 Faculty of Medicine, Cairo University, Giza, Egypt; 5 Institute of Pathology, Ruhr-University Bochum, Bochum, Germany

**Keywords:** Cholecystectomy outcomes, COVID-19, Patient behavior, Hospital logistics

## Abstract

**Background:**

Our study aimed to depict the influence of the COVID-19 pandemic on patients diagnosed with acute or chronic cholecystitis undergoing cholecystectomy.

**Methods:**

A retrospective cohort analysis was performed. The “Pre-pandemic cohort” (January 1, 2018, to March 21, 2020) was compared with the “Pandemic cohort” (March 22, 2020, to December 31, 2021), with March 22 marking the first national lockdown in Germany. A total of 1,075 patients were included (512 pre-pandemic, 563 during the pandemic). Demographic, clinical, perioperative, and postoperative variables were assessed. Descriptive and inferential statistical tests, including the Shapiro-Wilk and Mann-Whitney U tests, were applied.

**Results:**

Demographic profiles remained comparable between groups. Time from symptom onset to hospital presentation rose markedly (1.83 ± 4.75 vs. 8.49 ± 30.63 days, *p* < 0.001), while hospital workflow efficiency remained unaffected. Emergency procedures (25.4% vs. 31.1%, *p* = 0.02) and cases of acute cholecystitis (29.1% vs. 42.1%, *p* = 0.048) increased during the pandemic. Laparoscopic cholecystectomy remained the standard approach, though its relative frequency declined somewhat during the pandemic (89.9% vs. 81.9%), while open procedures tripled (4.2% vs. 13.5%, *p* < 0.01). Postoperative complication rates rose modestly (11.9% vs. 16.0%, *p* = 0.055), but did not reach statistical significance.

**Conclusion:**

The COVID-19 pandemic significantly altered patient behavior and clinical presentation, leading to more advanced disease and changes in surgical strategy. Despite increased case complexity, hospital logistics and critical outcomes remained robust, underscoring the adaptability of surgical teams to overcome the challenges posed by systemic healthcare crises. Remarkably, our study demonstrates that even in the shadow of a global pandemic, surgical practice does not stand still—strategies shift, workflows adapt, and outcomes endure, offering reassurance that future disruptions can be approached with confidence rather than concern.

**Supplementary Information:**

The online version contains supplementary material available at 10.1186/s12893-025-03445-z.

## Background

Gallstones affect approximately 20% of the European population, according to data from the European Association for the Study of the Liver (EASL) [1, 2]. Up to 50% of affected individuals develop symptoms over their lifetime. Cholecystitis is therefore one of the most prevalent gastrointestinal conditions in Europe. More than 175,000 cholecystectomies are performed annually for cholecystolithiasis in Germany [3].

Symptomatic patients are generally recommended to undergo cholecystectomy to relieve symptoms and prevent serious complications such as biliary pancreatitis, which remains associated with considerable morbidity and mortality. In cases of acute cholecystitis, urgent cholecystectomy within 24–72 h is recommended [4]. Delayed surgery can result in higher postoperative morbidity, prolonged hospital stays, and increased healthcare costs [5, 6].

In December 2019, the SARS-CoV-2 virus spread globally. While most infected individuals experience mild, flu-like symptoms, severe cases can develop acute respiratory distress syndrome (ARDS) requiring intensive care and mechanical ventilation [7]. The rapid escalation of COVID-19 cases led the German government to implement nationwide lockdown measures beginning March 22, 2020. Despite these measures, infection rates continued to rise in waves, with temporary declines during the summers of 2020 and 2021 [6, 8].

During the pandemic, intensive care resources and operating room capacities were reallocated to prioritize COVID-19 patients and those with malignant diseases. Elective procedures were postponed worldwide due to resource constraints [9]. Additionally, many patients avoided medical facilities out of fear of contracting the virus.

This study aimed to assess the impact of the COVID-19 pandemic on the surgical care and outcomes of patients with acute and symptomatic chronic cholecystitis undergoing cholecystectomy. We hypothesized that patient avoidance of medical facilities led to delayed presentation, resulting in higher morbidity, increased emergency operations, higher intraoperative conversion rates, more advanced histological stages, longer waiting times for surgery, and extended postoperative hospital stays, as discussed in previous studies [10–15].

## Methods

### Study design

This study was approved by the ethics committee of the Ruhr-University Bochum (Nr. 23–7778) and conducted following the Helsinki Declaration. Informed consent for surgery, data collection, and analysis was obtained from all patients.

### Patients

Between January 2018 and December 2021, 1,092 patients underwent cholecystectomy at our institution. Patients aged 18 and older who underwent cholecystectomy for acute or symptomatic (chronic) cholecystitis were included in the study. Seventeen patients did not meet these criteria and were excluded (Benign and malignant Gall bladder disease). Clinical and pathological findings, time from the first symptoms to hospitalization, time from hospitalization to surgery, operative time, surgical outcomes, and complications were retrospectively analyzed. The date of the first lockdown in Germany (March 22, 2020) was used as the cutoff for comparison. Patients operated on between January 1, 2018, and March 21, 2020, were classified as the “pre-pandemic Group.1” Patients operated on between March 22, 2020, and December 31, 2021, were classified as the “pandemic Group.2”, as per the study flow chart.

### Data collection

Patient and operative data were retrieved from individual patient records and compiled into a Microsoft Excel file. All surgical procedures were performed by experienced general surgeons (> 150 operations per year) following a standardized surgical technique [16]. Postoperative Outcomes were reported and complications were classified according to the Clavien-Dindo classification [17]. Mortality was defined as any death occurring during hospitalization or within 90 days post-surgery. Complete and detailed pathological reports were available for all patients.

### Statistical analyses

All statistical analyses were performed using SPSS software, version 29.0.0.0 (SPSS Inc., Chicago, IL, USA). Descriptive frequency analysis was performed to confirm the completeness of the dataset. The normality of continuous data distributions was assessed using the Shapiro-Wilk test and the Kolmogorov-Smirnov test. Continuous variables were summarized as medians with interquartile ranges (IQR), while categorical variables were reported as absolute numbers and percentages.

For non-normally distributed continuous data, comparisons between the pre-pandemic and pandemic groups were conducted using the Mann-Whitney U test. Categorical variables were compared using the chi-squared test for independence. Statistical significance was defined as a *p*-value ≤ 0.05.

## Results

A total of 1,075 patients underwent cholecystectomy during the study period, comprising 512 individuals in the pre-pandemic cohort and 563 in the pandemic cohort. The demographic and clinical characteristics of these patients are summarized in Table 1. Overall, the baseline characteristics were largely comparable between the two groups, with some notable exceptions reflecting potential shifts in patient selection and clinical presentation during the COVID-19 pandemic. Gender and age distributions were comparable across both groups, with a consistent female predominance (61.5% vs. 63.1%, *p* = 0.60) and a stable majority aged 35–64 years (50.8% vs. 51.7%, *p* = 0.43). The proportion of elderly patients (≥ 80 years) remained unchanged at around 10%.

**Table 1:** Patient characteristics — demographics, ASA class, BMI, risk factors, and preoperative presentation

		Pre-pandemic		Pandemic		*P*-value
		*N* = 512	%	*N* = 563	%	
Gender	male	197	38,5%	208	36,9%	0,6
	female	315	61,5%	355	63,1%	
ASA Classification	ASA 1	0	0,0%	1	0,2%	< 0.001
	ASA 2	387	76,2%	374	66,4%	
	ASA 3	122	23,8%	188	33,4%	
	ASA 4	0	0,0%	0	0,0%	
BMI	Normal weight	85	16,6%	67	11,9%	< 0.001
	Overweight	125	24,4%	78	13,9%	
	1 st degree obesity	90	17,6%	55	9,8%	
	2nd degree obesity	27	5,3%	15	2,7%	
Age	18–34	75	14,7%	71	12,6%	0,43
	35–64	260	50,8%	291	51,7%	
	65–79	128	25,0%	142	25,2%	
	80+	49	9,6%	59	10,5%	
Alcohol		13	2,5%	17	3,0%	0.63
Smoking		58	11,3%	44	7,8%	0.049
Preoperative pain		469	91,6%	450	79,9%	< 0.001
Preoperative jaundice		7	1,4%	10	1,8%	0.59
Pancreatitis	acute	50	9,8%	44	7,8%	0,25
	chronic	4	0,8%	9	1,6%	
Preoperative stenting CBD		59	11,5%	69	12,3%	0,71

This table summarizes baseline characteristics of patients undergoing cholecystectomy before and during the COVID-19 pandemic. Significant differences were observed in ASA classification, BMI distribution, smoking status, and prevalence of preoperative pain, indicating a higher perioperative risk profile and symptom severity in the pandemic cohort. All other demographic and clinical variables were comparable between groups

A significant increase in ASA-III patients during the pandemic (23.8% to 33.4%, *p* < 0.001) suggests a shift toward higher-risk surgical candidates, possibly due to delayed care or prioritization protocols. Smoking prevalence decreased (11.3% to 7.8%, *p* = 0.04), while alcohol use remained stable. Preoperative pain was reported less frequently during the pandemic (91.6% vs. 79.9%, *p* < 0.001), possibly indicating delayed presentation.

Perioperative findings and surgical parameters differed between the two cohorts, as summarized in Table 2. The rate of emergency cholecystectomies increased significantly during the pandemic, rising from 25.4% (*n* = 130) in the pre-pandemic group to 31.1% (*n* = 175) in the pandemic group (*p* = 0.02). Correspondingly, the intraoperative assessment revealed a shift in disease presentation. Chronic cholecystitis was more commonly observed before the pandemic (70.9%, *n* = 363), whereas acute cholecystitis was significantly more frequent during the pandemic period (42.1%, *n* = 237 vs. 29.1%, *n* = 149; *p* = 0.04).

**Table 2:** Perioperative findings and surgical data before and during the COVID-19 pandemic

		Pre-pandemic		Pandemic		*P*-value
		*N* = 512	%	*N* = 563	%	
Emergency operation		130	25,4%	175	31,1%	0.02
Intraoperative finding	chronic cholecystitis	363	70,9%	326	57,9%	0.048
	acute cholecystitis	149	29,1%	237	42,1%	
Surgical approach	laparoscopic	460	89,9%	461	81,9%	˂0.01
	conversion	30	5,9%	26	4,6%	
	open	22	4,2%	76	13,5%	
Histology	chronic cholecystitis	365	71,3%	348	61,6%	0.04
	Acute cholecystitis	147	28,7%	215	38,2%	
		Pre-Pandemic		During Pandemic		P-Value
		Median	SD	Median	SD	
Time from symptom onset to hospital presentation (days)		1.83	± 4.75	8.49	± 30.63	< 0.001
Time from admission to operation (days)		2.14	± 3.11	1.76	± 2.62	0.178
Operation time (minutes)		94.11	± 40.32	91.22	± 36.51	0.221

This table summarizes key operative and intraoperative parameters. During the pandemic, emergency procedures and acute cholecystitis presentations increased significantly, while laparoscopic cholecystectomy rates declined with a corresponding rise in open operations (*p* < 0.01). Time from symptom onset to hospital presentation was markedly prolonged (*p* < 0.001), reflecting delayed healthcare access. Operation duration and time from admission to surgery showed no statistically or clinically significant differences between groups

*SD* Standard deviation

Regarding the surgical approach, laparoscopic cholecystectomy remained the standard in both groups but was performed less frequently during the pandemic (81.9%, *n* = 461) compared to the pre-pandemic period (89.9%, *n* = 460). Notably, the rate of primary open cholecystectomy increased markedly during the pandemic (13.5%, *n* = 76) compared to the pre-pandemic phase (4.2%, *n* = 22), a statistically significant difference (*p* < 0.01). The conversion rates from laparoscopic to open surgery remained relatively low and comparable between the two periods (5.9% vs. 4.6%). Histopathological analysis confirmed the intraoperative findings, with chronic cholecystitis being more prevalent in the pre-pandemic group (71.3%, *n* = 365) and acute cholecystitis more common in the pandemic cohort (38.2%, *n* = 215 vs. 28.7%, *n* = 147; *p* = 0.04).

Operative logistics remained consistent across the two study periods, as per Table 2. The median time from hospital admission to surgery remained stable between groups (SD ± 3.0 vs. ±2.9; *p* = 0.4), showing no statistically significant difference. Similarly, the median operative duration showed no significant difference, with 94 min recorded pre-pandemic and 91 min during the pandemic (SD ± 40 vs. ±36; *p* = 0.22). These findings suggest that surgical efficiency and workflow were well maintained despite the broader strain imposed by the COVID-19 pandemic.

Postoperative complication rates increased modestly during the pandemic, rising from 11.9% (95% CI 9.1–14.7) to 16.0% (95% CI 12.9–19.1), although this trend did not reach statistical significance (*p* = 0.055) and should therefore be interpreted with caution. Notably, several complication subtypes classified by the Dindo-Clavien grading system showed significant increases. Grade I complications (including pneumonia) rose from 0.6% to 2.5% (*p* = 0.01), Grade 3 A (including percutaneous Drainage) from 0.6% to 1.6% (*p* = 0.01), and Grade 3B (including revision operations) from 0.6% to 1.2% (*p* = 0.02), indicating a shift toward more interventional postoperative management during the pandemic period.

Severe complications (Grades IV, including MOF) remained infrequent and showed no significant difference between groups (*p* = 0.91 and *p* = 0.89), reflecting a stable trend in severe outcome rates. Other postoperative events showed no statistically significant differences. Postoperative mortality increased slightly during the pandemic (1.4% vs. 0.8%), suggesting a non-significant trend rather than a definitive difference (*p* = 0.31), as shown in Table 3.

**Table 3:** Postoperative outcomes before and during the COVID-19 pandemic

		Pre-pandemic		Pandemic		*P*-value
		Count *N* = 512	%	Count *N* = 563	%	
Complications	No	451	88,1%	473	84,0%	0.055
	Yes	61	11,9%	90	16,0%	
Dindo-Clavien	No	451	88,1%	473	84,0%	0.055
	grade 1	3	0,6%	14	2,5%	0.01
	grade 2	45	8,8%	53	9,4%	0.06
	grade 3B	3	0,6%	7	1,2%	0.02
	grade 3 A	3	0,6%	9	1,6%	0.01
	grade 4	3	0,6%	3	0,5%	0.91
	grade 5	4	0,8%	4	0,7%	0.89
Delayed gastric emptying		3	0,6%	1	0,2%	0.27
postoperative bleeding		1	0,2%	0	0,0%	0.29
Wound healing problems		5	1,0%	10	1,8%	0.26
Re-operation		6	1,2%	5	0,9%	0.64
Mortality		4	0,8%	8	1,4%	0.31
ICU days		4	0,8%	7	1,2%	0.07

This table presents postoperative complication rates and their severity graded according to the Clavien–Dindo classification. Overall complications showed a trend toward increase during the pandemic (16.0% vs. 11.9%, *p* = 0.055), though this did not reach statistical significance. Notably, minor complications (grades I–IIIa) were slightly more frequent during the pandemic, whereas severe complications and mortality remained comparable between groups. These findings suggest that while patient and system factors during the pandemic modestly affected postoperative recovery, major outcomes were largely preserved

**Fig. 1:**
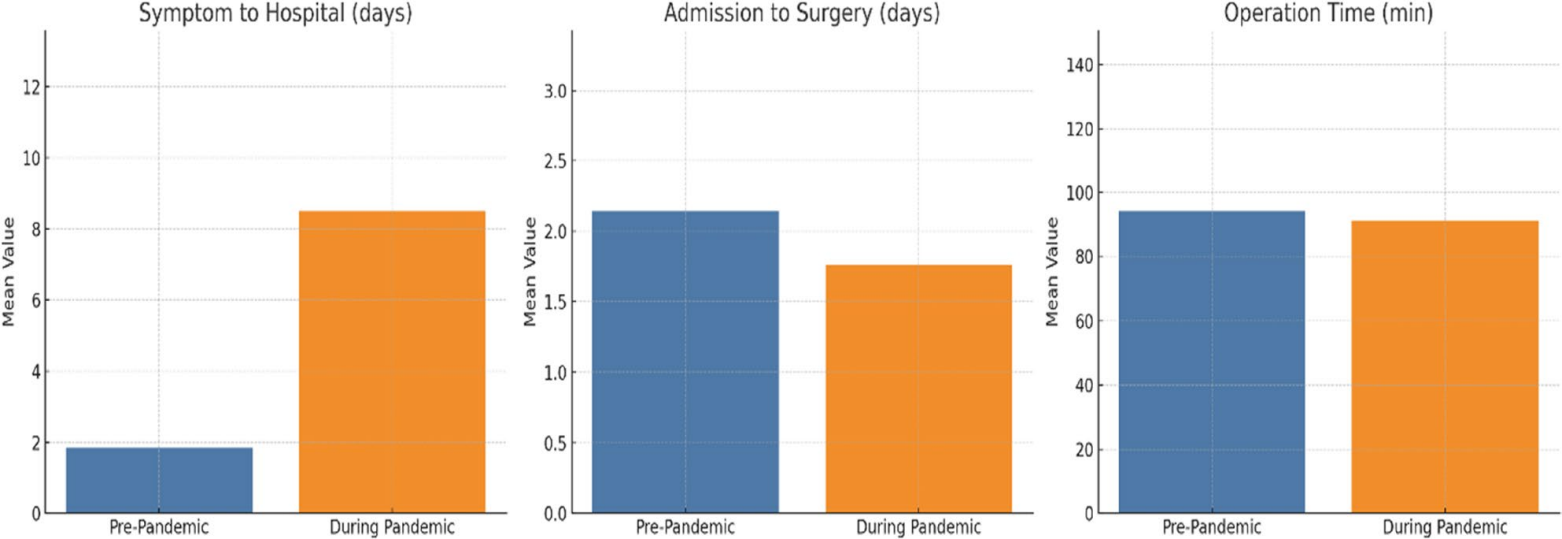
Key surgical metrics before vs. during the COVID-19 pandemic. Comparison of presentation delay (Symptom to Hospital time in days), (Admission to Surgery time in days), operative time in minutes, across periods

Table 2 compares clinical timing and operative data of the study’s patients. Statistically significant differences were found only in the time from symptom onset to hospital presentation (*P* < 0.001), reflecting altered patient behavior during the pandemic, as per Fig. 1. While the time from hospital admission to surgery (2.14 ± 3.1 days before the pandemic and 1.76 ± 2.6 days afterward) remained stable between the two groups, there was a marked increase in the delay from symptom onset to hospital presentation during the pandemic (a mean of 8.49 ± 30.63 days during the pandemic, compared to 1.83 ± 4.75 days pre-pandemic, *p* < 0.001). Operative time showed no statistically or clinically significant difference between groups (94.11 ± 40.32 vs. 91.22 ± 36.51 min, *p* = 0.2). However, the extended delay in the initial presentation highlights a potential shift in patient behavior during the pandemic period.

**Fig. 2:**
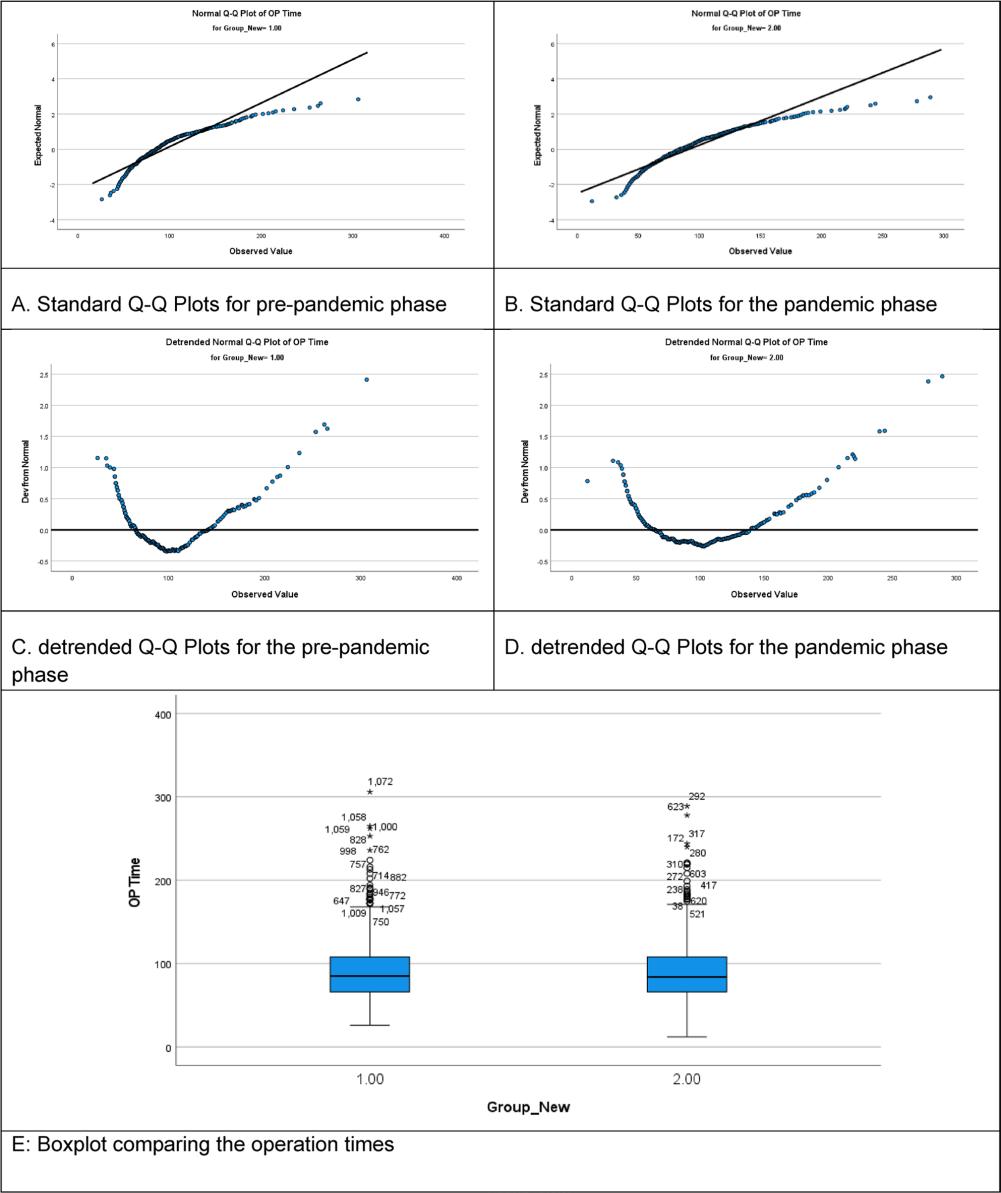
Operation-time distribution and normality diagnostics (multi-panel). **A** Standard Q–Q plot (pre-pandemic): operation times show mild tail deviations. **B** Standard Q–Q plot (pandemic): distribution closely approximates normality with fewer extreme values. **C** Detrended Q–Q plot (pre-pandemic): highlights greater dispersion and tail outliers.**D** Detrended Q–Q plot (pandemic): reduced deviation suggests more standardized workflows. **E** Boxplot of operation time by period: similar medians/IQRs with fewer outliers during the pandemic

Figure 2A-D. demonstrates the normal and detrended Q–Q plots for operation time, providing a visual assessment of the distributional characteristics. Figure 2E. presents a boxplot comparing the operation times of cholecystectomies across the two study periods. The figure demonstrates that both the median operation time and interquartile ranges (IQRs) were comparable between the pre-pandemic and pandemic groups, indicating that the central tendency and overall distribution of surgical duration remained stable despite the challenges posed by the COVID-19 pandemic.

The pre-pandemic group (Group 1) shows a greater number of outliers, with some operation times exceeding 300 min, reflecting a broader variability in surgical duration. In contrast, the pandemic group (Group 2) exhibits fewer and less extreme outliers, implying a more standardized or streamlined operative workflow during this time. This reduction in extreme values may be attributable to modified surgical protocols, enhanced resource management, or prioritization of efficiency in response to the constraints imposed by the COVID-19 pandemic.

**Fig. 3:**
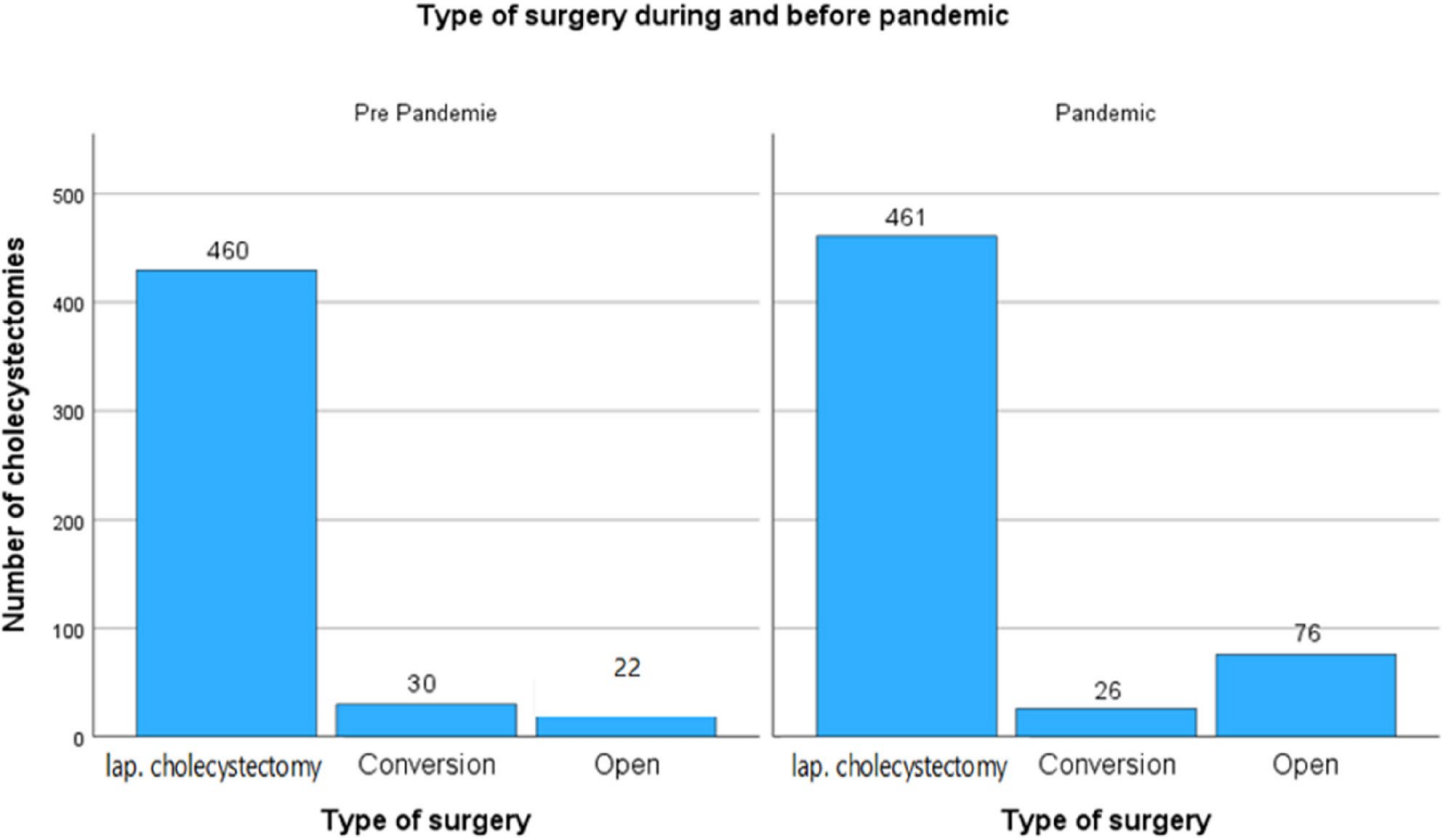
Surgical approach before and during the COVID-19 pandemic. A modest shift toward open cholecystectomy was observed during the pandemic, reflecting adjustments in case selection and intraoperative decision-making in response to logistical and infection-control challenges

Figure 3 displays a grouped bar chart comparing the distribution of surgical approaches used for the cholecystectomy operation. The three surgical categories—laparoscopic cholecystectomy, conversion to open surgery, and primary open cholecystectomy—are presented for both timeframes. In both periods, laparoscopic cholecystectomy remained the predominant approach. While the absolute number of laparoscopic procedures remained nearly unchanged, the relative proportion decreased during the pandemic, suggesting a shift in surgical practice or patient selection. The conversion rate from laparoscopy to open surgery showed a slight decline, from 30 cases pre-pandemic (5.9%) to 26 cases during the pandemic (4.6%). This minor decrease may reflect improved intraoperative decision-making or stricter selection criteria for laparoscopic candidates during the pandemic period.

A notable finding is the marked increase in primary open cholecystectomies, which rose from 22 cases (4.2%) in the pre-pandemic group to 76 cases (13.5%) during the pandemic. This more than threefold increase indicates a significant deviation from the minimally invasive standard and suggests several possible contributing factors. These may include a higher proportion of complicated or emergency cases, institutional concerns over laparoscopy in the pandemic, or efforts to expedite operative management under constrained conditions.

Collectively, this figure highlights a pandemic-associated trend toward a more pragmatic surgical approach, with increased reliance on open procedures and a modest reduction in minimally invasive surgery. These patterns underscore the broader impact of systemic pressures on operative strategy and resource utilization in acute care surgery.

## Discussion

This study aimed to investigate the impact of the COVID-19 pandemic on the clinical presentation, perioperative management, surgical approach, and outcomes of patients undergoing cholecystectomy. Through a comparative analysis of 1,075 patients—512 treated before and 563 during the pandemic—we sought to identify how shifts in patient behavior and systemic healthcare adaptations influenced the management of cholecystitis.

Our analysis revealed stable demographic distributions in both cohorts. Gender ratios were similar, with a consistent female predominance, and age distribution remained unchanged, with the majority of patients falling in the 35–64 age group. These findings align with prior studies, such as those by Koch et al. 2022 [13], and Blohm et al. 2023 [18], who reported that the demographic composition of surgical candidates remained stable during the pandemic, mostly due to the persistent and non-elective nature of symptomatic gallbladder disease.

However, a significant shift was observed in patient comorbidity profiles. The proportion of ASA III patients rose markedly during the pandemic (from 23.8% to 33.4%, *p* < 0.001), indicating a shift toward operating on patients with higher anesthetic and perioperative risk. This is consistent with findings by Cano-Valderrama et al. 2020 [19], who documented a prioritization of higher-risk or more urgent surgical candidates due to resource reallocation and cancellation of elective procedures. In contrast, Sandblom et al. 2022 [20] reported no significant changes in ASA classifications, which may reflect differences in healthcare system strain or triage policies.

A notable finding of our study was the significant delay in time from symptom onset to hospital presentation during the pandemic (mean 8.5 ± 30 vs. 1.83 ± 4.7 days, *p* < 0.001). This suggests that patients delayed seeking care, likely due to fear of hospital-acquired COVID-19, limited access to outpatient services, or restrictions imposed by lockdowns. Similar delays in presentation have been reported in multiple studies [6, 13, 18, 20], with some associating them with increased severity of intra-abdominal infections and worsened surgical outcomes.

Interestingly, the time from hospital admission to surgery remained stable (2.14 vs. 1.76 days, *p* = 0.4), and operative times were comparable (85 vs. 86 min, *p* = 0.97). These findings suggest that while patients arrived later, hospital logistics adapted effectively, maintaining timely surgical access. This contrasts with the findings by Vigneswaran et al. 2020 [21], who reported prolonged in-hospital delays due to COVID-related logistics. However, our results support a recent study [20], which also found that intra-hospital workflows were well preserved despite the external pressures of the pandemic. However, on a broader scale, our findings align with those of Mogharab et al. (2022) [22], who demonstrated in a global meta-analysis that pandemic-related delays significantly influenced surgical timing and outcomes, supporting the observed shifts in our cohort.

The increase in emergency surgeries (25.4% to 31.1%, *p* = 0.02) and higher incidence of intraoperative acute cholecystitis (29.1% to 42.1%, *p* = 0.048) during the pandemic underscores the clinical consequences of delayed presentation. These findings are corroborated by De Simone et al. 2025 [23], who reported a higher proportion of gangrenous and perforated gallbladders during the pandemic. Histopathological data in our study confirmed these trends, with acute cholecystitis diagnoses rising significantly (28.7% to 38.2%, *p* = 0.04). These results further highlight the shift from elective, less emergent cases to more urgent, inflammatory presentations.

Despite the rising complexity, laparoscopic cholecystectomy remained the primary surgical approach in both groups. However, its relative use declined during the pandemic (89.9% to 81.9%), with a concurrent and significant increase in primary open cholecystectomies (4.2% to 13.5%, *p* < 0.01). This shift may reflect institutional preferences for open access in complicated cases [21] or a pragmatic strategy to reduce operative time and complications in emergency settings.

The literature offers mixed perspectives on this trend. Di Saverio et al. [24] recommended a conservative stance on laparoscopy early in the pandemic, which aligns with our observed increase in open procedures. Conversely, Chadi et al., 2020 [25] and Hatampour et al., 2023 [26] reported that laparoscopic surgery remained safe and standard in institutions with well-established perioperative protocols, highlighting the variability of practice based on local policies and resources.

Interestingly, conversion rates from laparoscopy to open surgery remained stable (5.9% vs. 4.6%), suggesting that surgeon decision-making and intraoperative thresholds for conversion were consistent, and technical execution remained uncompromised. Similar findings were reported by Hatampour et al. 2023 [25].

Overall postoperative complications increased modestly during the pandemic (11.9% to 16.0%), although this difference approached but did not reach statistical significance. Notably, several Dindo-Clavien subgroups showed statistically significant increases: Grade I (0.6% to 2.5%, *p* = 0.01), Grade IIIA (0.6% to 1.6%, *p* = 0.01), and Grade IIIB (0.6% to 1.2%, *p* = 0.02). These findings indicate a trend toward more interventional or procedural management of complications, possibly reflecting more advanced disease at the time of surgery or heightened vigilance during postoperative care.

In contrast, severe complications—including MOF (Grade IV) and postoperative mortality—remained rare and unchanged. ICU admission and reoperation rates were also comparable, suggesting that although the clinical burden was elevated, the quality and safety of surgical care remained intact. These observations are consistent with those of Koch et al. [13], who found stable complication rates in experienced centers, and contrast with our previous study [6], which showed higher morbidity and ICU utilization in their pandemic cohort.

Figure 2 confirmed the consistency in operative timing between the two cohorts, with comparable medians and interquartile ranges. Notably, the pre-pandemic group exhibited a wider range and more outliers, with some procedures exceeding 300 min. In contrast, the pandemic group demonstrated a narrower distribution, possibly reflecting efforts to standardize workflows, limit resource use, or reduce exposure times. These subtle differences indicate that procedural efficiency may have improved despite systemic constraints, consistent with findings from other studies on surgical logistics during the pandemic [6, 13, 25].

A significant pandemic-associated trend toward more open procedures was demonstrated in Fig. 3. While laparoscopic cholecystectomy remained the standard technique, the increase in primary open surgeries represents a clear deviation from standard practice. This likely reflects the higher acuity of cases, institutional concerns about laparoscopy in COVID-positive or untested patients, and broader shifts in surgical strategy during crisis periods.

To date, no meta-analysis has comprehensively addressed the specific perioperative parameters and system-related changes associated with cholecystectomy during the COVID-19 pandemic. Recognizing this gap, our research group has recently initiated a systematic review and meta-analysis to synthesize data from a broad range of studies. This effort seeks to more accurately quantify the impact of the COVID-19 pandemic on surgical outcomes, patient behavior, and healthcare system logistics related to cholecystectomy procedures.

While our findings highlight distinct shifts in patient presentation and surgical strategy, these results should also be viewed in the context of institutional and logistical adaptations during the pandemic. Changes such as altered operating room allocation, prioritization of emergency and high-risk cases, and modified perioperative workflows likely influenced both the timing and outcomes of cholecystectomy procedures. Furthermore, evolving infection control measures, staff shortages, and resource redistribution may have contributed to subtle variations in surgical practice and postoperative care [27, 28]. Recognizing these contextual factors enables a more balanced interpretation of the observed trends and underscores the adaptability of surgical systems in crisis conditions.

### Strengths and limitations

This study benefits from a large, real-world cohort spanning two distinct periods and offers a comprehensive evaluation of clinical, logistical, and procedural variables. The chronological organization of the analysis allows for clear temporal comparisons, and the use of objective outcome measures (e.g., the Dindo-Clavien classification) strengthens data interpretation.

However, several limitations must be acknowledged. As a single-center, retrospective study, it is subject to inherent biases in documentation and case selection. We could not assess long-term outcomes or quality-of-life data, and factors such as vaccination status, COVID-19 positivity, or changes in surgical staffing were not captured.

Although this study provides a large and comprehensive overview of cholecystectomy practice during the pandemic, several unmeasured confounders may have affected the results. The timing and extent of the COVID-19 vaccination rollout could have influenced both patient presentation patterns and hospital admission policies, potentially modifying surgical volumes and outcomes. Similarly, the emergence of new viral variants with differing transmission dynamics may have shaped institutional responses and patient behaviors over time. Finally, staffing shortages, altered shift structures, and resource redistribution within hospitals during the pandemic might have contributed to variations in perioperative care and complication rates. While these factors were not directly analyzed, acknowledging their influence adds important context to the interpretation of our findings.

Another limitation of this study is the absence of a multivariate analysis to adjust for potential confounders such as ASA classification, patient age, and surgical urgency. Although the large cohort and standardized protocols provide a consistent framework for comparison, unmeasured variations in these parameters may have influenced the observed outcomes and should be considered when interpreting the results.

Lastly, generalizability may be limited, as institutional protocols and regional pandemic severity vary widely.

## Conclusion

This study highlights the multifaceted impact of the COVID-19 pandemic on the management of cholecystitis. The COVID-19 pandemic significantly altered patient behavior and clinical presentation, leading to more advanced disease and changes in surgical strategy. Despite increased case complexity, hospital logistics and critical outcomes remained robust, underscoring the adaptability of surgical teams to overcome the challenges posed by systemic healthcare crises. Remarkably, our study demonstrates that even in the shadow of a global pandemic, surgical practice does not stand still—strategies shift, workflows adapt, and outcomes endure, offering reassurance that future disruptions can be approached with confidence rather than concern, as effective surgical care continues to evolve in response to emerging challenges. The insights gained may help shape future protocols for managing surgical care during global health emergencies.

## Supplementary Information

Supplementary Material 1.

## Data Availability

The datasets generated and/or analyzed during the current study are not publicly available due to institutional data protection policies but are available from the corresponding author upon reasonable request. Please contact Dr. Ahmed Abdelsamad (Email: ahmed6361410@gmail.com).

## Abbreviations

ICU: Intensive care unit
EASL: Association for the study of the liver
MOF: Multiple organ failure
SD: Standard deviation
