# Supplementary material for: Cholecystectomies in the shadow of COVID-19 pandemic: a retrospective analysis of 1075 patients – shift in patient behavior, hospital logistics, and perspectives for the future
Source: BMC Surg. 2025 Dec 23;26:74. doi: 10.1186/s12893-025-03445-z (PMC12836890; doi:10.1186/s12893-025-03445-z)
Supplement: Supplementary file 1 — Supplementary Material 1. [file 12893_2025_3445_MOESM1_ESM.doc]

**Study Flow Diagram**

**Allocation**

**Analysis**

**Follow-Up**

**Enrollment**

Assessed for eligibility (n=1092) )

Excluded (n= 17 )

♦  Not meeting inclusion criteria (n= 17 )

♦  Declined to participate (n= 0 )

♦  Other reasons (n= 0 )

Pre-pandemic phase G1 (n= 512 )

♦ Received allocated intervention (n= 512 ) (Cholecystectomy operations)

Lost to follow-up (give reasons) (n= 0 )

Discontinued intervention (give reasons) (n= 0)

Pandemic phase G2 (n= 563 )

♦ Received allocated intervention (n= 563 ) (Cholecystectomy operations)

Analysed (n= 1075 )
♦ Excluded from analysis (give reasons) (n= 0)

Included (n= 1075 )
